# Supplementary figures and images for: Two predicted models based on ceRNAs and immune cells in lung adenocarcinoma
Source: PeerJ. 2021 Mar 23;9:e11029. doi: 10.7717/peerj.11029 (PMC7996073; doi:10.7717/peerj.11029)

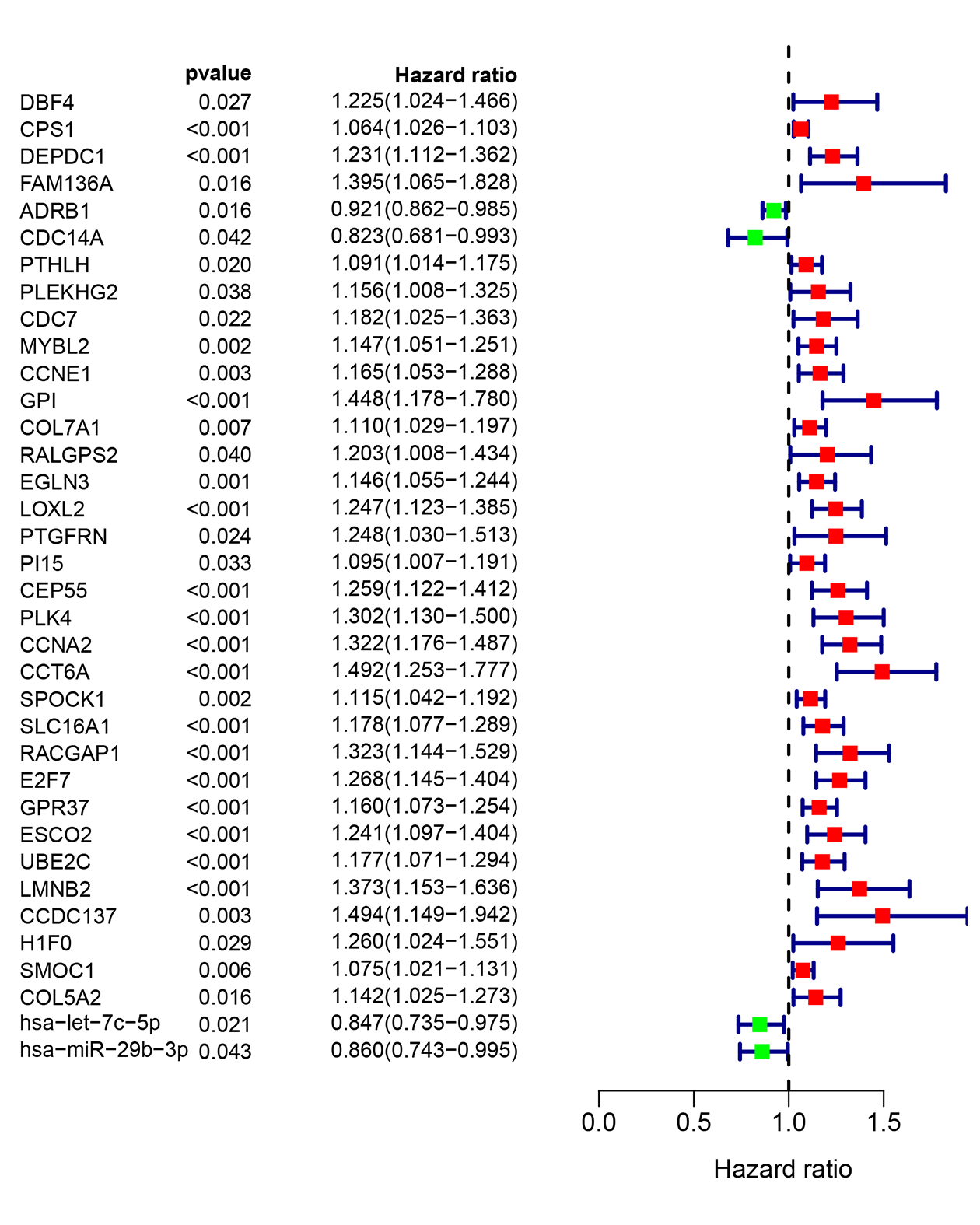

Supplement: Figs. S1 [file peerj-09-11029-s001.png]

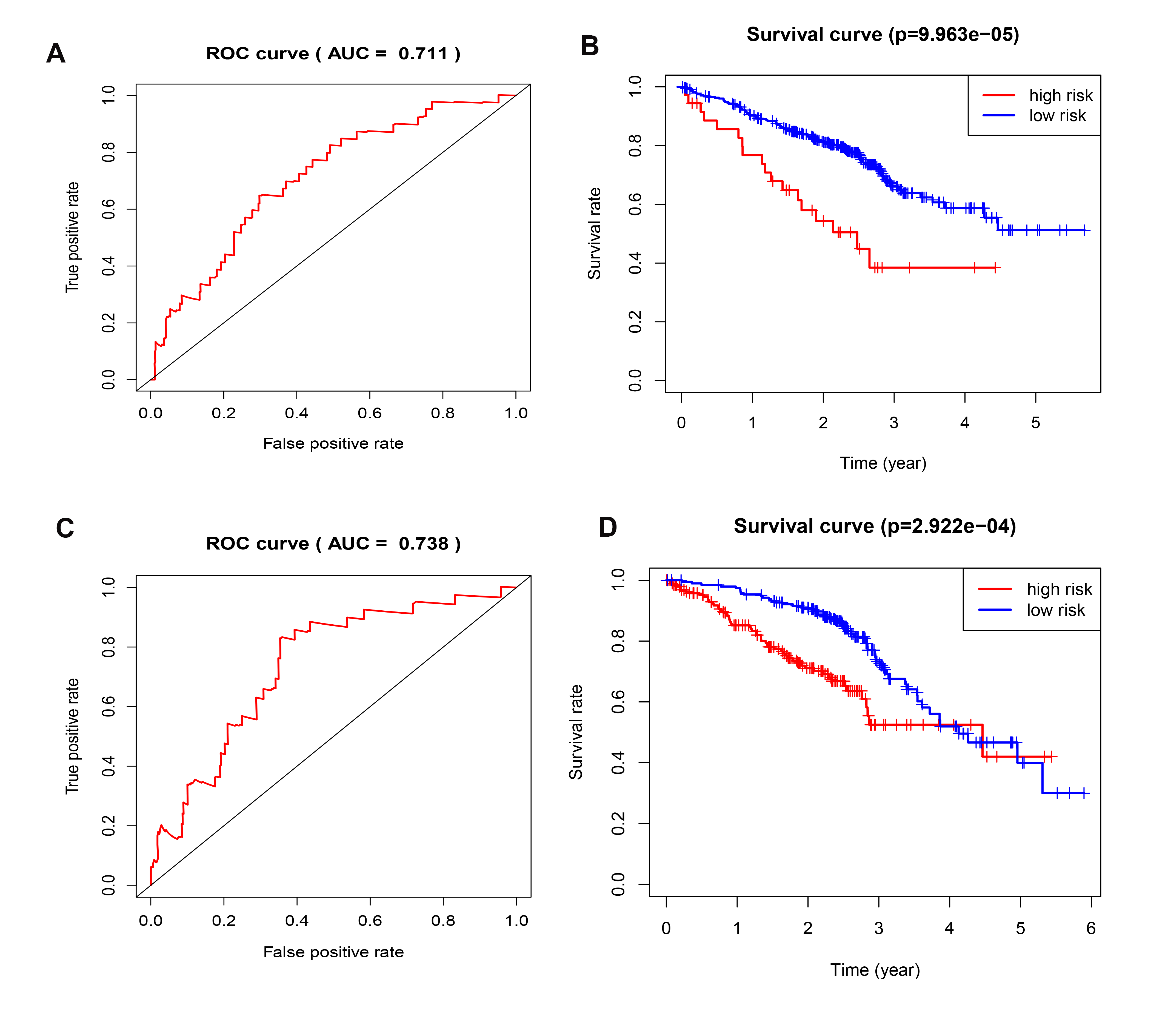

Supplement: Figs. S2 — (A), (B) K-M curve and ROC curve of ceRNAs signatures. (C), (D) K-M curve and ROC curve of immune cells signatures. [file peerj-09-11029-s002.png]

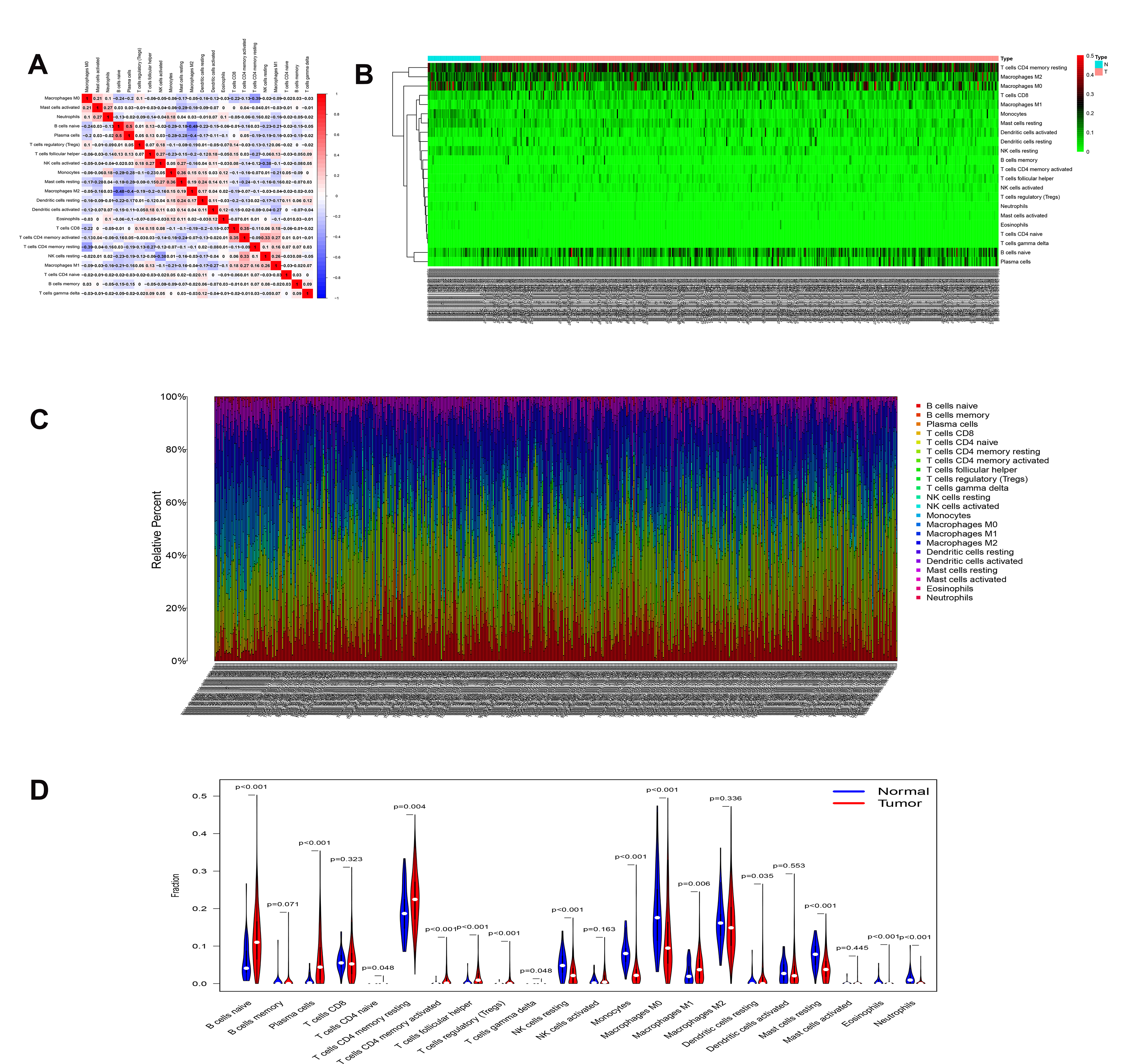

Supplement: Figs. S3 — The correlation analysis of immune cells (A).The heatmaps (B) and composition (C) of 22 immune cell types estimated by CIBERSORT. The violin plot of immune cells (D). The red bar and blue bar represent tumor group and normal group, respectively. [file peerj-09-11029-s003.png]

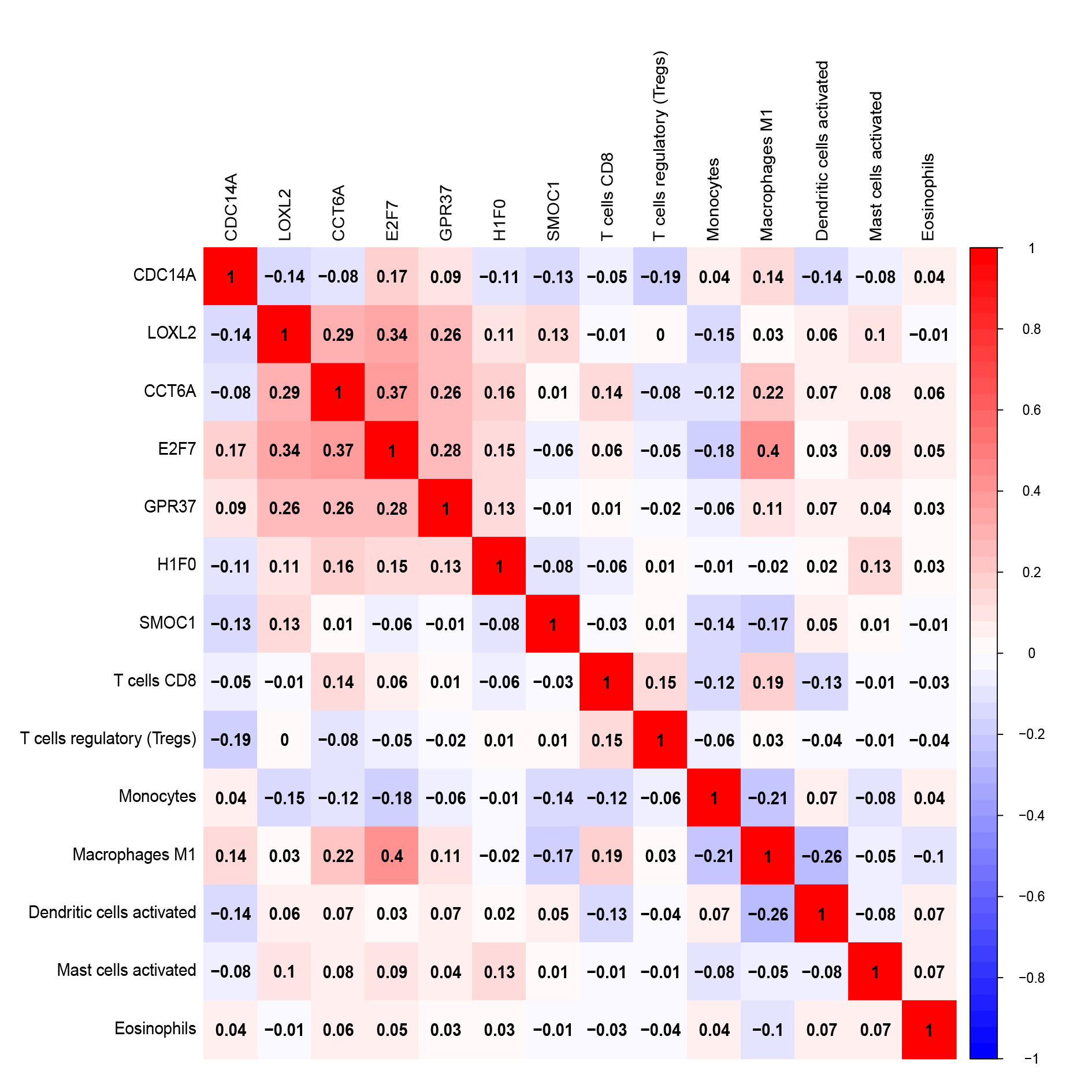

Supplement: Fig. S4 [file peerj-09-11029-s004.png]

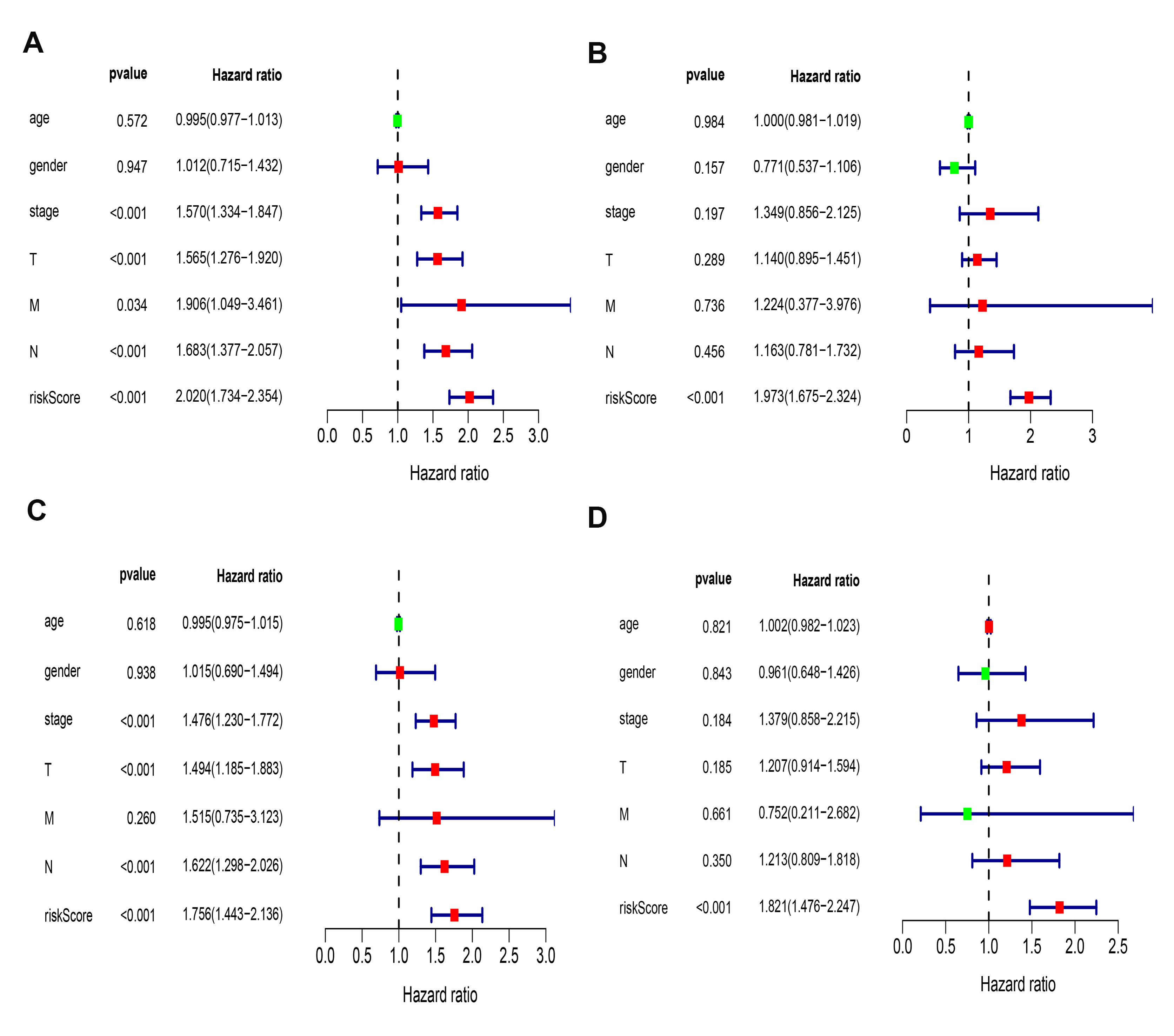

Supplement: Figs. S5 — Univariate analysis (A) and multivariate analysis (B) between the ceRNA-based prognostic model and clinicopathologic factors. Univariate analysis (C) and multivariate analysis (D) between the immune-cell-based prognostic model and clinicopathologic factors. [file peerj-09-11029-s005.png]

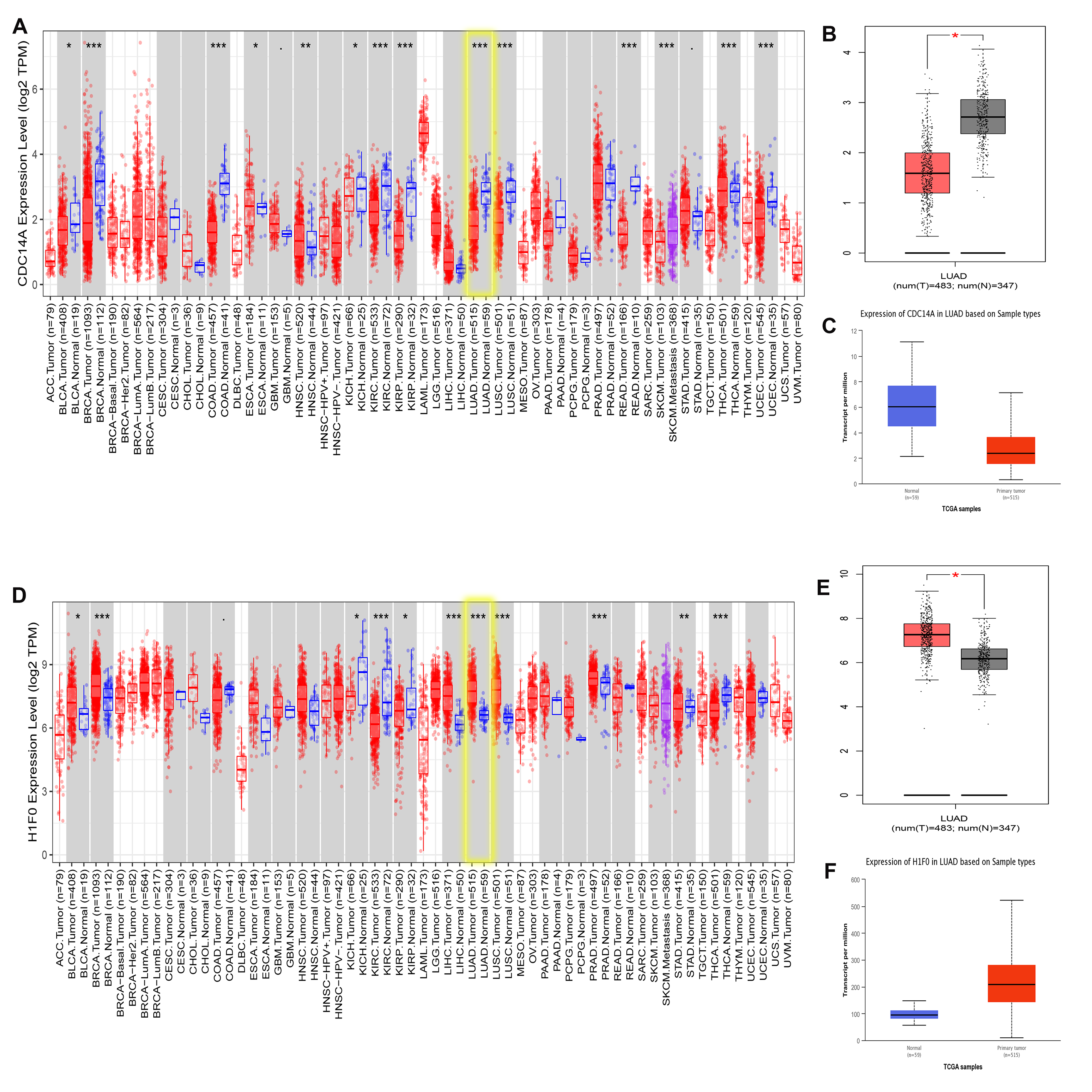

Supplement: Figs. S6 — (A), (B), (C) validation expression of CDC14A. (D), (E), (F) validation expression of H1F0. [file peerj-09-11029-s006.png]

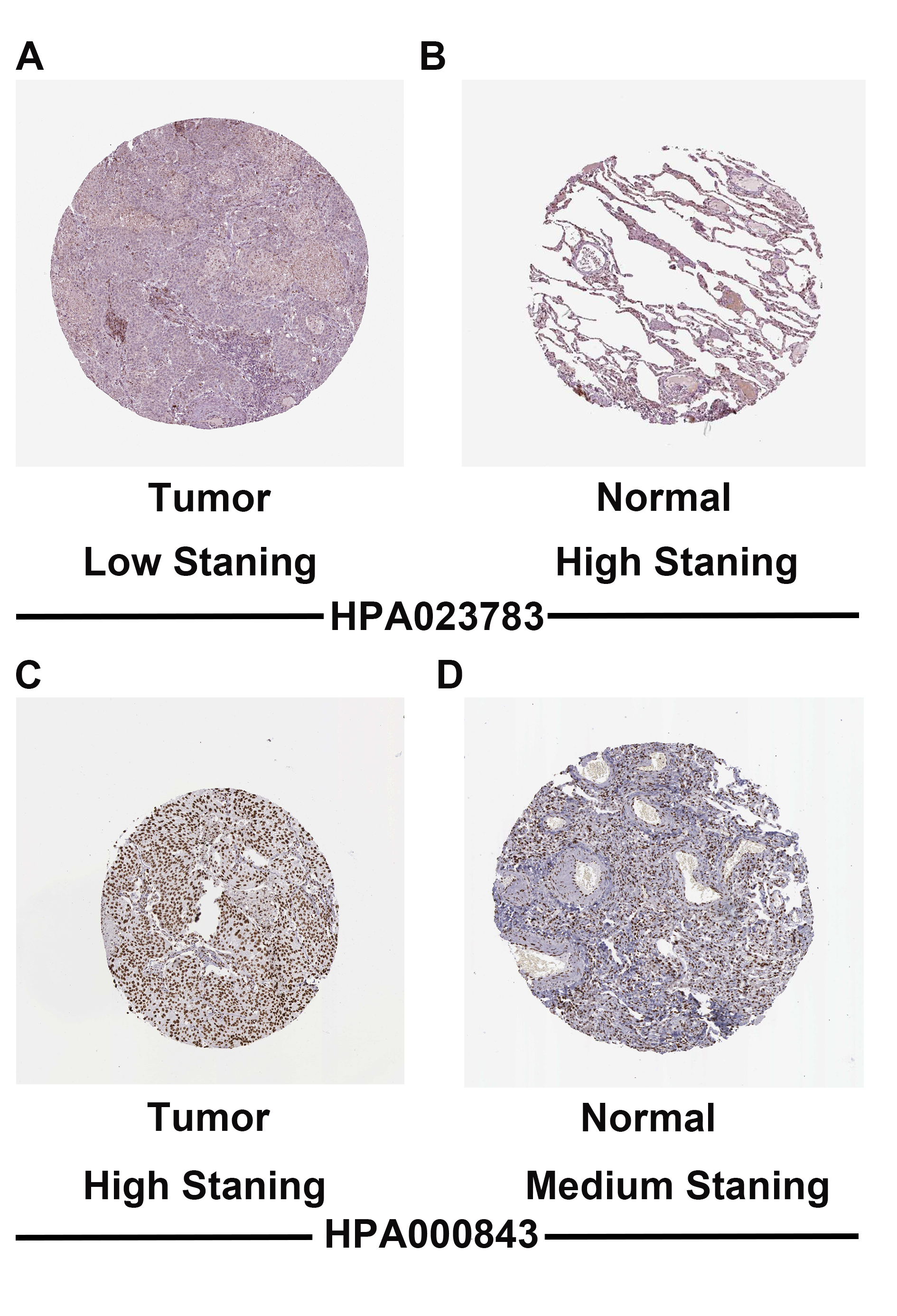

Supplement: Fig. S7 — (A), (B) CDC14A protein expression between normal and tumour tissues. (C), (D) H1F0 protein expression between normal and tumour tissues. HPA, The Human Protein Atlas data. [file peerj-09-11029-s007.png]

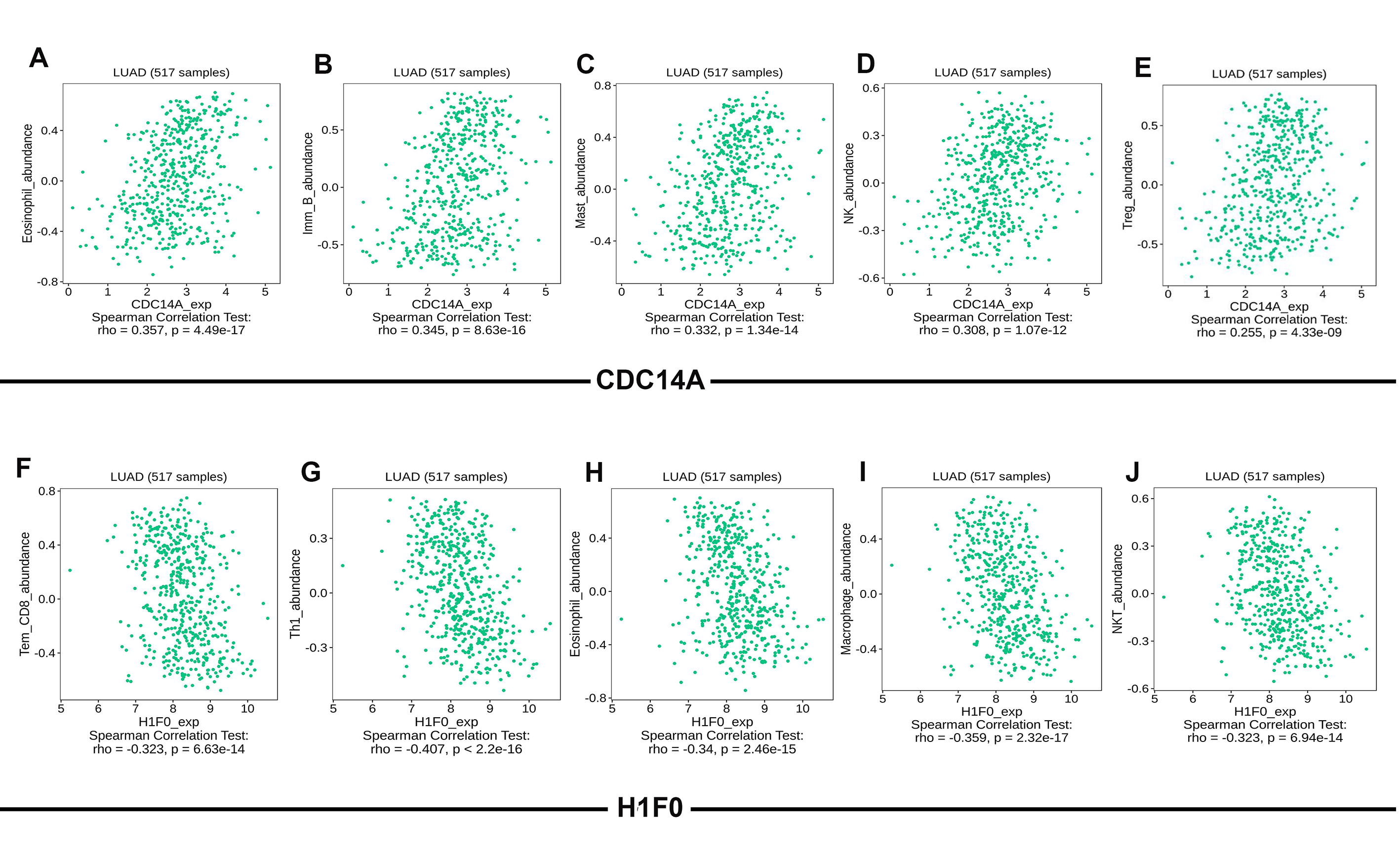

Supplement: Fig. S8 [file peerj-09-11029-s008.png]

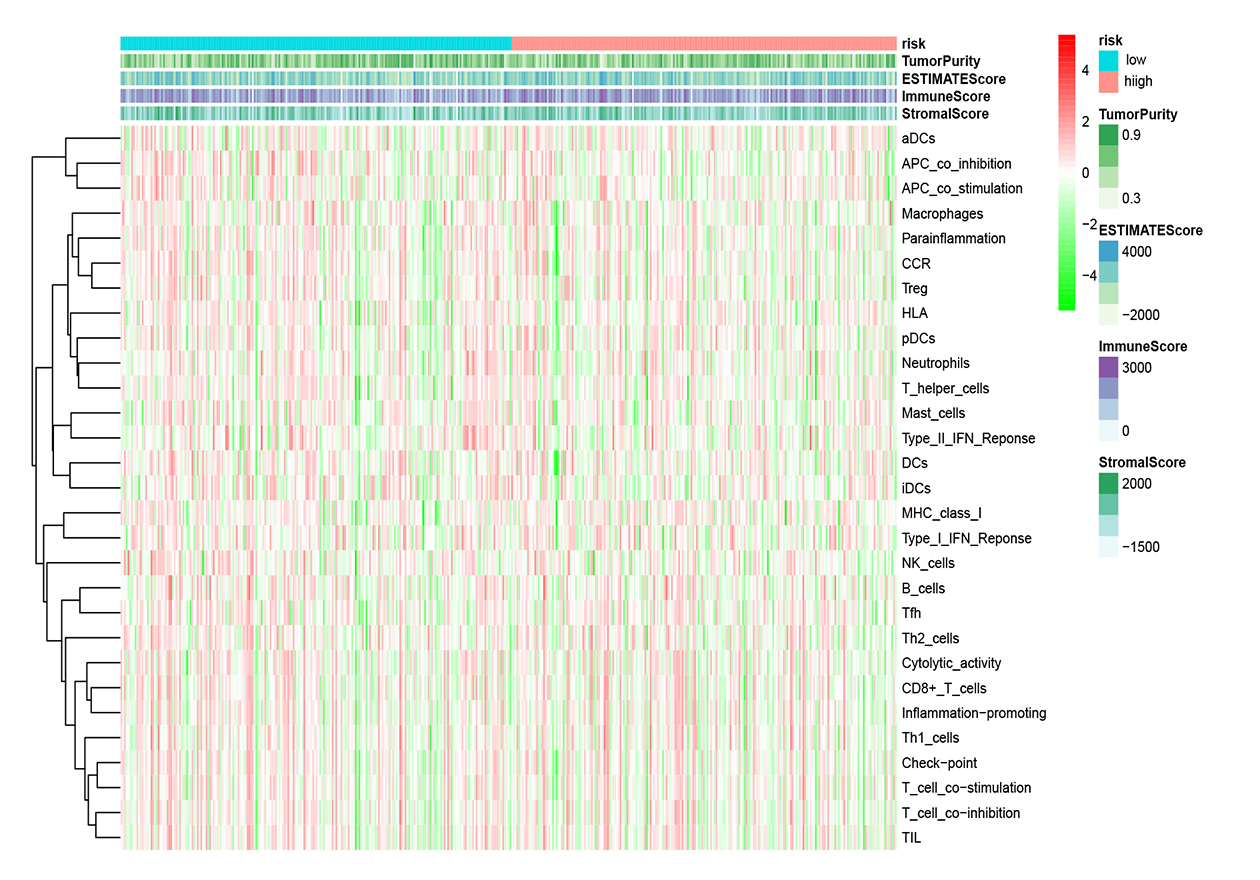

Supplement: Fig. S9 [file peerj-09-11029-s009.png]
