# Supplementary material for: Two predicted models based on ceRNAs and immune cells in lung adenocarcinoma
Source: PeerJ. 2021 Mar 23;9:e11029. doi: 10.7717/peerj.11029 (PMC7996073; doi:10.7717/peerj.11029)
Supplement: Table S2 [file peerj-09-11029-s011.doc]

**Supplementary Table 2 Hypergeometric testing and correlation analysis of ceRNAs network**

| lncRNA | mRNA | miRNA | Coef | Cor P | hyper P |
| --- | --- | --- | --- | --- | --- |
| PVT1 | TMEM182 | hsa-miR-17-5p | 0.169 | 4.76E-05 | 0.050 |
| PVT1 | MRPL24 | hsa-miR-20a-5p,hsa-miR-93-5p | 0.380 | 8.40E-20 | 0.011 |
| SNHG1 | PITX2 | hsa-miR-21-5p | 0.180 | 1.65E-05 | 0.010 |
| SNHG1 | RALGPS2 | hsa-miR-326,hsa-miR-21-5p | 0.101 | 0.010 | 0.005 |
| SNHG1 | GPI | hsa-miR-326 | 0.200 | 1.91E-06 | 0.001 |
| SNHG1 | LMNB2 | hsa-miR-326 | 0.252 | 2.23E-09 | 0.002 |
| SNHG1 | NETO2 | hsa-miR-21-5p | 0.175 | 2.76E-05 | 0.041 |
| SNHG1 | ANKRD13B | hsa-miR-326 | 0.245 | 6.30E-09 | 0.041 |
| MAGI2-AS3 | ADRB1 | hsa-miR-374b-5p,hsa-miR-374a-5p | 0.256 | 1.29E-09 | 0.033 |
| MAGI2-AS3 | CYBRD1 | hsa-miR-374b-5p,hsa-miR-374a-5p | 0.578 | 1.08E-48 | 0.013 |
| MAGI2-AS3 | CDC14A | hsa-miR-374b-5p,hsa-miR-374a-5p | 0.369 | 1.06E-18 | 0.007 |
| MAGI2-AS3 | PARD6B | hsa-miR-374b-5p,hsa-miR-374a-5p | 0.092 | 0.017 | 0.033 |
| MAGI2-AS3 | ID4 | hsa-miR-374b-5p,hsa-miR-374a-5p | 0.278 | 4.13E-11 | 0.016 |
| MAGI2-AS3 | MEIS1 | hsa-miR-374b-5p,hsa-miR-374a-5p | 0.492 | 8.70E-34 | 0.005 |
| MAGI2-AS3 | TTLL7 | hsa-miR-374b-5p,hsa-miR-374a-5p | 0.106 | 0.007 | 0.004 |
| MAGI2-AS3 | DST | hsa-miR-374b-5p,hsa-miR-374a-5p | 0.373 | 4.25E-19 | 0.001 |
| MAGI2-AS3 | LRCH2 | hsa-miR-374b-5p,hsa-miR-374a-5p | 0.431 | 1.43E-25 | 0.011 |
| MAGI2-AS3 | NOVA1 | hsa-miR-374b-5p,hsa-miR-374a-5p | 0.076 | 0.040 | 0.004 |
| SNHG3 | PITX2 | hsa-miR-340-5p | 0.210 | 5.43E-07 | 0.029 |
| SNHG3 | GPT2 | hsa-miR-340-5p | 0.203 | 1.37E-06 | 0.036 |
| SNHG3 | RCC1 | hsa-miR-340-5p | 0.256 | 1.22E-09 | 0.011 |
| SNHG3 | PLK4 | hsa-miR-340-5p | 0.107 | 0.007 | 0.004 |
| SNHG3 | PUS7 | hsa-miR-340-5p | 0.207 | 7.86E-07 | 0.029 |
| SNHG3 | DBF4 | hsa-miR-340-5p | 0.132 | 0.001 | 0.022 |
| SNHG3 | H1F0 | hsa-miR-340-5p | 0.093 | 0.017 | 0.011 |
| SNHG3 | SLFN13 | hsa-miR-340-5p | 0.108 | 0.007 | 0.011 |
| SNHG3 | SKP2 | hsa-miR-340-5p | 0.150 | 0.000 | 0.004 |
| AC074117.1 | E2F2 | hsa-let-7a-5p, hsa-let-7c-5p,hsa-let-7b-5p | 0.355 | 2.07E-17 | 0.000 |
| FBXL19-AS1 | PDIA4 | hsa-miR-378a-3p | 0.148 | 0.000 | 0.005 |
| FBXL19-AS1 | CLK2 | hsa-miR-378a-3p | 0.359 | 9.91E-18 | 0.044 |
| FBXL19-AS1 | PLEKHG2 | hsa-miR-378a-3p | 0.332 | 2.76E-15 | 0.001 |
| H19 | UBE2C | hsa-miR-138-5p | 0.191 | 4.88E-06 | 0.043 |
| H19 | DEPDC1 | hsa-miR-130b-3p | 0.165 | 7.32E-05 | 0.011 |
| H19 | CEP55 | hsa-miR-130b-3p | 0.157 | 0.000 | 0.017 |
| H19 | GPT2 | hsa-miR-130b-3p | 0.185 | 9.26E-06 | 0.006 |
| H19 | MYBL2 | hsa-miR-29b-3p | 0.226 | 8.01E-08 | 0.003 |
| H19 | SASH1 | hsa-miR-130b-3p | 0.209 | 6.27E-07 | 0.003 |
| H19 | PEAK1 | hsa-miR-29b-3p | 0.088 | 0.022 | 0.021 |
| H19 | NDST1 | hsa-miR-29b-3p | 0.231 | 3.87E-08 | 0.005 |
| H19 | CCNA2 | hsa-miR-130b-3p | 0.138 | 0.001 | 0.047 |
| H19 | CCNE1 | hsa-miR-138-5p | 0.099 | 0.012 | 0.021 |
| H19 | HEG1 | hsa-miR-130b-3p | 0.166 | 6.59E-05 | 0.004 |
| H19 | SEMA6A | hsa-miR-138-5p | 0.173 | 3.12E-05 | 0.014 |
| H19 | ESCO2 | hsa-miR-130b-3p | 0.119 | 0.003 | 0.001 |
| H19 | FAM136A | hsa-miR-29b-3p | 0.168 | 5.22E-05 | 0.006 |
| H19 | TRAF4 | hsa-miR-29b-3p | 0.077 | 0.038 | 0.001 |
| H19 | E2F2 | hsa-miR-130b-3p | 0.227 | 7.28E-08 | 0.025 |
| H19 | SOX4 | hsa-miR-138-5p,hsa-miR-130b-3p | 0.214 | 3.68E-07 | 0.037 |
| H19 | CPS1 | hsa-miR-29b-3p | 0.191 | 5.16E-06 | 0.001 |
| H19 | COL1A1 | hsa-miR-29b-3p | 0.186 | 8.95E-06 | 0.004 |
| H19 | TSPAN18 | hsa-miR-130b-3p | 0.117 | 0.004 | 0.011 |
| H19 | PDK1 | hsa-miR-138-5p,hsa-miR-130b-3p | 0.140 | 0.001 | 0.005 |
| H19 | RAPGEF4 | hsa-miR-130b-3p | 0.082 | 0.030 | 0.004 |
| H19 | E2F7 | hsa-miR-29b-3p,hsa-miR-130b-3p | 0.221 | 1.42E-07 | 0.001 |
| H19 | EGLN3 | hsa-miR-130b-3p | 0.270 | 1.44E-10 | 0.025 |
| H19 | SIX4 | hsa-miR-130b-3p | 0.145 | 0.000 | 0.023 |
| H19 | ZFYVE9 | hsa-miR-130b-3p | 0.175 | 2.70E-05 | 0.001 |
| H19 | RNF122 | hsa-miR-29b-3p | 0.134 | 0.001 | 0.006 |
| H19 | KDM5B | hsa-miR-29b-3p | 0.133 | 0.001 | 0.025 |
| H19 | NHSL1 | hsa-miR-130b-3p | 0.145 | 0.000 | 0.002 |
| H19 | COL7A1 | hsa-miR-29b-3p | 0.255 | 1.40E-09 | 0.001 |
| H19 | CCDC137 | hsa-miR-130b-3p | 0.179 | 1.73E-05 | 0.021 |
| H19 | COL5A2 | hsa-miR-29b-3p | 0.215 | 3.28E-07 | 0.000 |
| H19 | TTYH3 | hsa-miR-130b-3p | 0.235 | 2.53E-08 | 0.001 |
| H19 | RFLNB | hsa-miR-29b-3p | 0.219 | 2.00E-07 | 0.005 |
| H19 | FIBIN | hsa-miR-130b-3p | 0.086 | 0.024 | 0.002 |
| H19 | LRCH1 | hsa-miR-130b-3p | 0.108 | 0.006 | 0.020 |
| H19 | FZD4 | hsa-miR-130b-3p | 0.161 | 0.000 | 0.025 |
| H19 | SOX12 | hsa-miR-29b-3p | 0.193 | 4.11E-06 | 0.001 |
| H19 | SMOC1 | hsa-miR-130b-3p | 0.186 | 9.01E-06 | 0.002 |
| H19 | SPOCK1 | hsa-miR-130b-3p | 0.312 | 1.06E-13 | 0.041 |
| H19 | GPR37 | hsa-miR-29b-3p | 0.110 | 0.006 | 0.001 |
| H19 | HOXA5 | hsa-miR-130b-3p | 0.127 | 0.002 | 0.007 |
| H19 | SLC16A14 | hsa-miR-29b-3p | 0.198 | 2.30E-06 | 0.011 |
| H19 | RACGAP1 | hsa-miR-130b-3p | 0.157 | 0.000 | 0.025 |
| H19 | PTGFRN | hsa-miR-130b-3p | 0.222 | 1.36E-07 | 0.014 |
| H19 | TCF4 | hsa-miR-29b-3p, hsa-miR-138-5p,hsa-miR-130b-3p | 0.102 | 0.010 | 0.000 |
| H19 | PTHLH | hsa-miR-29b-3p | 0.260 | 6.58E-10 | 0.004 |
| H19 | DIO2 | hsa-miR-29b-3p | 0.139 | 0.001 | 0.005 |
| H19 | CCT6A | hsa-miR-130b-3p | 0.182 | 1.30E-05 | 0.017 |
| H19 | OTUB2 | hsa-miR-29b-3p | 0.085 | 0.025 | 0.002 |
| H19 | NPTX1 | hsa-miR-130b-3p | 0.129 | 0.002 | 0.014 |
| H19 | CDC7 | hsa-miR-29b-3p | 0.171 | 3.90E-05 | 0.008 |
| H19 | LOXL2 | hsa-miR-29b-3p | 0.255 | 1.40E-09 | 0.002 |
| H19 | ANKRD13B | hsa-miR-29b-3p,hsa-miR-138-5p | 0.236 | 1.99E-08 | 0.003 |
| H19 | AKT3 | hsa-miR-29b-3p | 0.316 | 5.62E-14 | 0.011 |
| H19 | MLLT11 | hsa-miR-29b-3p | 0.308 | 2.50E-13 | 0.001 |
| H19 | HMGCS1 | hsa-miR-29b-3p | 0.141 | 0.001 | 0.014 |
| H19 | MYBL1 | hsa-miR-130b-3p | 0.074 | 0.044 | 0.006 |
| H19 | TUBB2A | hsa-miR-29b-3p | 0.096 | 0.014 | 0.000 |
| H19 | KIF26A | hsa-miR-29b-3p | 0.133 | 0.001 | 0.002 |
| H19 | KIAA1211 | hsa-miR-130b-3p | 0.188 | 7.06E-06 | 0.002 |
| H19 | PI15 | hsa-miR-29b-3p | 0.159 | 0.000 | 0.002 |
| H19 | HOXB3 | hsa-miR-130b-3p | 0.167 | 5.65E-05 | 0.005 |
| H19 | MAP2K6 | hsa-miR-29b-3p | 0.126 | 0.002 | 0.003 |
| H19 | PRKAA2 | hsa-miR-130b-3p | 0.140 | 0.001 | 0.035 |
| H19 | COL4A5 | hsa-miR-29b-3p | 0.102 | 0.010 | 0.047 |
| H19 | SLC16A1 | hsa-miR-29b-3p | 0.192 | 4.60E-06 | 0.011 |
